# Methods of assessing and recording bladder sensation: a review of the literature

**DOI:** 10.1007/s00192-018-3760-x

**Published:** 2018-09-05

**Authors:** Hayser Medina Lucena, Douglas G. Tincello

**Affiliations:** Department of Health Sciences, University of Leicester, Centre for Medicine, University Road, Leicester, Leicestershire LE1 7RH UK

**Keywords:** Urinary bladder, Humans, Urodynamics, Focus groups, Urinary incontinence

## Abstract

**Introduction and hypothesis:**

The objective was to review different methods that have been used to assess bladder sensation and to provide an overview of the accuracy and objectivity of the measurement of the subjective perception of the bladder.

**Methods:**

The MEDLINE and PubMed databases were searched to identify articles. References from those articles were also searched. Terms used for the search were: urinary bladder, sensation, cystometry, urodynamics, urinary incontinence and focus group. Eight hundred and fifty abstracts were identified from databases, and 12 from other sources. Twenty-two duplicate articles were removed. Irrelevant articles were excluded after reading their titles. Fifty-four articles were eligible, but 17 were excluded after reading the full text, leaving 37 articles where assessment of bladder sensation was the main aim.

**Results:**

Six different methods of measuring bladder sensation have been described in the literature. Although the most frequently used was cystometry, this is an invasive tool and does not reproduce bladder behaviour during daily life because it records bladder sensation as episodic events. The visual analogue scale using a forced diuresis protocol seemed to be an excellent tool. It was non-invasive and evaluated bladder sensation continuously, from an empty to a full bladder.

**Conclusions:**

In some of the studies, the samples were too small to draw any significant conclusions. There were also conflicting data on which tool was the most accurate, especially as each method of evaluating bladder sensation may influence the way it is described by participants.

## Introduction

Bladder sensation occurs as a result of the relationship between volume and pressure, detected by pressure and stretch receptors within the bladder wall. Determining its pattern in healthy people is challenging, because there is a dearth of evidence on how individuals perceive bladder sensation and how they respond to different stimuli. The sensory pathway that processes bladder perception is complex. Various intrinsic [1] and extrinsic factors have influenced this pathway, such as abdominal pressure, bowel status, posture and hydration, but these are yet to be confirmed [2, 3].

Sensation-related bladder diaries (SR-BDs) [4–7], cystometry [8–10], visual analogue scales (VAS) [11, 12], frequency volume charts [13] and forced diuresis [2, 14] are some of the tools used to measure bladder sensation. Although bladder sensation develops as a continuous and increasing process, most of these methods assess bladder perception by asking for three separate events: first sensation of filling (FSF), first desire to void (FDV) and strong desire to void (SDV) [15]. Episodic events do not correlate with the way in which the sensation develops [16].

Although lately, there has been more interest in objective and non-invasive methods of measuring bladder sensation, there is still no consensus on which tool is appropriate. The aim of this article is to provide an overview of methods used to measure bladder sensation and which one is the most accurate and objective for measuring the subjective perception of the bladder.

## Materials and methods

The MEDLINE and PubMed databases were searched to identify articles for this literature review. Reference lists from those articles were also searched for additional references. Only articles in English were reviewed. The terms used to for the search were: urinary bladder, sensation, cystometry, urodynamics, urinary incontinence and focus group. The search identified 850 abstracts from the MEDLINE and PubMed databases, and 12 from other sources from 1930 to 2017. Twenty-two duplicate articles were removed. Irrelevant articles were excluded after reading their titles. Although 54 articles were eligible on abstract screening, 17 were excluded after reading the full text as bladder sensation was not the main outcome. We focused on 38 articles where assessment of bladder sensation was the main aim.

## Results

### Invasive test used to measure bladder sensation

Denny-Brown and Robertson were amongst the first to focus on sensation during cystometry in 1933 [17]. It has been considered the most common tool for assessing bladder awareness, although it is invasive and requires artificial filling [13]. Some studies have proved that cystometry is a reliable and reproducible test [8–10, 18, 19]. The first was published in 1992 [8]. Ten patients underwent the test twice in the same sitting and 95 underwent the tests 15 months apart. Patients were asked to report their bladder sensations spontaneously. The study found that 80% of the short-term participants and 73% of the long-term group had an identical pattern of filling sensation: FSF, FDV and SDV. The FSF was the most underreported sensation. Similarly, in 2002, Wyndaele and De Wachter [19] compared the bladder sensation between two groups of volunteers who underwent cystometry 5 years apart. All participants had the same sequence of bladder sensation: FSF, FDV and SDV. In 2011, Van Meel and Wyndaele [18] confirmed these findings. The cystometries were repeated with an interval of 7 days in 13 volunteers and 17 patients with overactive bladder (OAB). They found that all participants followed a similar pattern of sensation, with a strong correlation between the degrees of sensation during each week in all subjects (correlation coefficient *R* > 0.5).

In 2004, Erdem et al. [9] challenged the reliability of this test in 59 patients with lower urinary tract symptoms (LUTS). The cystometry comprised three phases: in phases one and three, no infusion was pumped, and in phase two, normal saline was pumped. In phase two, all patients reported sensations within the same pattern. In the phases of no infusion, 47 (79.6%) reported an FSF, 38 (64.4%) reported FDV, and 15 (25.4%) reported SDV. It does appear that the same pattern of bladder sensation is seen, even with a lack of filling. This suggests that cystometry is not reproducible and, the false sensation can be associated with having the catheter in situ rather than actual filling.

Subsequently, Erdem et al. [20] conducted another study to investigate whether catheters had an impact on bladder sensation. Forty-five patients underwent cystometry in three phases. In the first phase the catheter was not inserted, in the second phase the catheter was inserted but no infusion was given and in the third, bladder filling was performed. In the latter, all sensations were perceived. When comparing the two first phases, none of the patients in phase one reported SDV and a higher percentage of patients felt the three sensations (FSF 86%, FDV 53% and SDV 17%) in phase two. These results were similar to the numbers found in their previous study [9]. It does appear, therefore, that urethral catheters and bladder filling can affect patients’ sensations, as a considerable number of patients perceived FSF and FDV even without a catheter.

De Wachter et al. tested the reliability of cystometry by blinding the patients to a real and a sham bladder filling: no medium was infused [10]. Fifty-nine patients with LUTS were recruited and, in contrast to Wyndaele’s study [8], none had any neurogenic condition. Similar results to previous studies were obtained during a real cystometry: 88% of patients were able to identify the same pattern of bladder sensation [10]. However, during sham cystometry, the findings differed from Erdem’s results: a small number reported sensations (5 FSF, 1 FDV), but none reported SDV. Together, these studies demonstrate that bladder filling and the presence of a catheter elicit similar sensations, but that these can be reported by patients even in the absence of infusion or a catheter. Thus, it appears that the subjective nature of these artificial definitions is open to suggestion by the environment of the test, or the staff performing it.

The publication of these studies highlights that there are conflicting data regarding the reliability of cystometry. We agree with De Wachter et al. [10] that cystometry cannot be considered unreliable just because some sensations are reported during sham cystometry. The urinary catheter can play an important role in the perception of bladder sensation, but other factors such as memory, habituation and artificial environment can also influence it.

### Non-invasive methods and cystometry used to measure bladder sensation

#### Frequency–volume chart and cystometry

A frequency–volume chart provides information regarding voided volume, 24-h urine production and frequency. De Wachter added a grading system to measure bladder sensation and then compared it with cystometry [13]. In this study, 15 volunteers were asked to fill out the chart and score the bladder perception before each void from 0 to 4, 0 corresponding to no sensation, 1 to FSF, 2 to FDV, 3 to SDV and 4 to an urgent desire to void (UDV) that cannot be deferred. Participants were encouraged to void for each sensation. The study compared bladder volumes at the time of each sensation on frequency–volume charts to cystometry and found only small differences in the volumes measured (*p* > 0.2) [13]. It does appear, therefore, that this tool can be used in the evaluation of bladder perception, and has the potential advantage of being non-invasive. Further, it also allows recording of bladder sensation during a physiological “normal” bladder filling. However, the small sample size here should be noted.

#### Visual analogue scales and cystometry

Visual analogue scales (VAS) are easy to use and reliable [11]. Dompeyre et al. applied this scale for the first time to assess bladder sensation [12]. This VAS was a 10-cm horizontal scale, with the left extremity meaning total absence of any desire and the right, an irresistible desire to void. The 25 patients underwent two consecutive cystometries: one using the standard method and the other using the VAS, in which each level of sensation was recorded, together with the bladder volume at which it occurred. The results revealed that the number of distinct levels (median of 9) on the VAS exceeded the three predetermined levels of the standard method (FSF, FDV and SDV). In 18 of the 25 cases, the graphic representation of the distinctive levels from the VAS revealed a linear and progressive curve. The first phase corresponded to the period of latency between no sensation and FSF. The second phase was a linear increase from the first sensation until the desire to void emerged. When the VAS definitions were compared with the standard cystometry definitions for first sensation and maximum capacity, there was a strong correlation (coefficient > 0.9) between the mean volumes at FSF (mean difference was 26.3 ml, *p* = 0.242) and at maximum cystometric capacity (MCC; mean difference of 3.8 ml, *p* = 0.716).

This was the first study where bladder sensation was described as a continuous phenomenon. These findings differed from those of other studies [17, 21], where bladder sensation was described as episodic in nature.

#### Sensation-related bladder diary and cystometry

The SR-BD is a 3-day bladder diary in which patients report sensations and volumes at which the grade of desire to void occurs. Grade 1 corresponded to no desire to void, 2 as FDV, 3 as SDV and 4 as a UDV. In 2009, Naoemova et al. [5] used this tool for the first time to compare bladder sensation with cystometry. One hundred and eighty-five patients filled out the SR-BD and underwent cystometry. As reported in other studies [4, 10, 13, 18, 19, 21], this study found a significant correlation between increasing bladder volumes and increasing degree of sensation in SR-BD (*p* < 0.02) and each desire to void during cystometry (*p* < 0.0004).

In contrast to De Wachter’s study [13], where voided volumes at each sensation reported in frequency–volume charts were comparable with the volumes at each sensation during cystometry, Naoemova et al. [5] found that patients described the degree of desire to void at larger volumes when undergoing cystometry than when completing the SR-BD. For example, patients with stress urinary incontinence reported FDV at 206 ml during SR-BD and 325 ml during cystometry.

#### Urgeometer and cystometry

The urgeometer is a keypad that patients can activate to measure the grade of urgency. Some studies have evaluated the use of this device to measure bladder sensation during cystometry [22–24].

In 2005, Craggs [24] asked 35 patients with OAB to use a five-level scoring urgeometer, from 0, meaning no sensation, to 5, being desperate. When comparing the mean bladder volumes at sensation scores 1, 2 and 3, they found that volumes increased significantly at each score (score 1 mean volume of 132.8 ml, score 2, 187.6 ml and score 3, 289.5 ml; *p* < 0.001).

In contrast, in 2007, Tsang et al. [23] asked 30 patients to use an urgeometer with four levels of urgency (first, mid, maximum and total urge volume, with a maximum score of 200). The test was repeated twice to assess reproducibility. The authors found that there was no difference in the values of urgency when comparing all tests, except at mid urge intensity (*p* = 0.024).

In 2008, Lowenstein et al. [22] assessed bladder sensation by using a different type of urgeometer. It has a sliding cursor that moves from the left “no urge” to the right “extreme urge to void”. Fifty-one patients reported their bladder sensation by moving the lever and also according to the International Continence Society (ICS) terms (FDV, SDV and MCC). When patients were analysed together, the mean urgeometer score increased significantly during the test: FDV (21 ± 18), SDV (68 ± 27) and MCC (85 ± 21), *p* < 0.001. They also compared the mean urgency levels between the groups (DO, USI and mixed) and found that it was higher in patients with DO (405 ml) than in the other groups (USI 500 ml or mixed 515 ml).

Analysis clearly showed that the urgeometer could be used as a different approach to assessing bladder sensation. It is simple and gathers information without the investigator’s intervention.

### Non-invasive methods used to measure bladder sensation

#### Sensation-related bladder diary

The SR-BD was compared in 251 incontinent women and 27 healthy volunteers. Volunteers had a different voiding pattern to patients. The latter group had increased urinary frequency (>2–3 voids/day; *p* < 0.002), lower bladder volumes (<100–300 ml/day; *p* < 0.0001) and increased bladder sensation (*p* < 0.0001 in grades 2 to 3). SR-BD also helped to distinguish differences between the various incontinent groups, i.e., the group with urge incontinence had the smallest mean voided volume and the highest urinary frequency [6].

If more days are added to a bladder diary, it can increase accuracy, but also reduce compliance [25]. For this reason, Naoemova et al. [7] conducted another study to assess its reliability, comparing results obtained from the SR-BD between the 3 days in 246 incontinent women. They found no difference in the 24-h voiding frequency, voided volumes or in the mean voided volumes at each grade of bladder sensation each day. The intraclass correlation (ICC) was calculated between mean voided volume and grade of sensation. The ICC varied between 0.20 and 0.80. The lower values were in voids with no inconvenience (grade 1). Higher ICC values were found with higher grades of desire (grade 2 to 4), which helped to conclude that the data from SR-BD seemed constant throughout different days.

The SR-BD appears to be a good tool. It provides the grade of sensation at each void, which could help to evaluate treatment. It could also help to distinguish between incontinent groups.

#### Forced diuresis

This method entails a water-load protocol, where individuals are asked to drink a large amount of water (1 l every hour) to achieve a fixed diuresis rate while recording their bladder sensation on an X/Y graph. The X-axis refers to a timescale and the Y-axis to intensity of sensation from 0 (no sensation) to 100 (absolute need to void). It is believed that by using non-invasive bladder filling and not relying on the use of words, there is minimal observer bias. The first study using this method included 11 healthy volunteers who underwent this protocol three times [14]. Each session ended when there was an absolute need to void. The diuresis rate did not vary between the three sessions (12.2 ± 3.4 ml/min). When describing the pattern of sensation graphically, two patterns were found (L and S shape). Both curves had a start with a slow rise, which corresponded to a gradual increase in intensity. The second phase showed a steep rise in intensity: for the L shape and in the S shape, there was a 3rd phase, where the sensation had a gradual increase before the need to void.

A similar study was conducted in 40 healthy women of different ethnic origins [2]. Bladder sensation was assessed after drinking plain water and artificially sweetened water. They found voided volumes were the same when comparing Asians with Caucasians (median 600 ml [300–1,200 ml]; *p* = 0.236) and also when comparing plain water and sweetened water groups (*p* = 0.450). In contrast to the De Wachter study [14], 16 women had a difference in mean diuresis rate of >5 ml/min; therefore, this group was excluded from the analysis to leave the rest with a similar value. They found that the time to achieve maximum bladder sensation was reduced when drinking sweetened water, although there was no effect on the bladder volume irrespective of ethnic group (35 min [20–85] vs 45 min [20–80], *p* < 0.001). This raises the question of whether by using this water-load protocol most of the participants achieve a constant diuresis rate or whether other factors such as a larger amount of fluid intake or different type of coaching need to be taken into consideration.

Most recently, Nagle et al. assessed real-time bladder perception using a combination of a forced diuresis protocol with a novel sensation meter [26], a tablet touch-screen surface, from 0% (empty bladder) to 100% (bladder fullness). In contrast to other studies, 14 normal and 12 OAB participants were asked to drink a commercial sport drink instead of water. However, the former may increase bladder sensitivity as it is known to be acidic [27]. Similar to other studies, maximum voided volumes were higher in volunteers than in patients. Sensations were perceived at lower volumes in OAB patients. The authors concluded that real-time sensation curves give a more comprehensive tool for assessing bladder filling, in agreement with others [14].

#### Focus groups

These can help to measure, determine or describe a particular topic. Two studies have used this tool in combination with the water-load protocol to assess bladder sensation [1, 28]. Both studies asked subjects to go through three repetitive focus groups and were asked to describe and locate their bladder sensation while undergoing a water-load protocol. The purpose was to reach a consensus on the terms used to portray bladder sensation. In the last session, all words were pooled, and participants had to choose from them. One study involved 11 volunteers [1] and another 10 patients with OAB [28]. The consensus words to describe bladder sensation (pressure and tingling) and the pattern (no sensation, weak awareness, stronger awareness, weak need, stronger need for an absolute need to void) were the same in both studies. The location of the sensation (abdomen or genitalia) varied from individual to individual. Patients with OAB described the sensation of pressure as continuous, and tingling as episodic. The results also showed that, in contrast with the volunteers, the volume at which the first sensation was felt was lower. Patients could also not delay voiding when the SDV appeared. When comparing bladder volume with intensity of sensation graphically, the patient group’s curve was steeper than the volunteers’ curve. These studies have shown how bladder sensation is experienced in healthy and in OAB patients without any influence from the investigator. Most importantly, it demonstrates that sensation can be perceived continuously rather than episodic, as proposed by the ICS [15]. On the other hand, the reported sensations using focus groups may not correlate with daily life symptoms.

#### VAS and SR-BD versus VAS and forced diuresis

A study of 26 volunteers and 12 OAB patients compared two non-invasive tools in two different settings. Participants filled in a VAS and a four-point urge scale in a bladder diary at home and then during natural forced diuresis at the hospital [29]. Although patients had a higher daily frequency and smaller voided volumes than patients, the perception of bladder fullness and degree of urge was directly related to bladder volume. Interestingly, both groups perceived the same bladder fullness level and urge score at higher volumes in the hospital setting than at home, i.e. bladder fullness of 10/10 was felt at 280 ml at home and at 500 ml in the hospital setting. These findings are comparable with Naoemova’s study, where the same degree of desire to void was felt at larger bladder volumes during cystometry, i.e. SDV was recorded in SR-BD at 291 ml, and at 419 ml during cystometry [5].

These findings reinforce what other studies have mentioned [14, 26, 28, 29]. VAS and SR-BD are effective non-invasive tools that help to assess bladder sensation, although this study adds that by using both tools together, the accuracy could be even better. For example, an urge scale of 4/4, bladder fullness sensation of 9/10 and a voided volume of less than half of the bladder capacity could indicate OAB.

## Discussion

Bladder sensation is a subjective symptom that is difficult to define, describe and measure. Any change in the perception of the bladder could signify a pathological condition of the lower urinary tract. As outlined, several patient-reported outcomes have been developed and put in place to try to measure bladder sensation. Most of the time, these methods are not comparable. Some studies use the same tool, but the scale, terms used to score it, or types of device used differ, which makes it difficult to compare and draw any definitive conclusions.

Cystometry is the method that is most frequently researched and has proved to be generally reliable [8, 10, 18, 19]. Some studies, however, still use different terms, which does not help when a comparison of data is required [9], although efforts have been made to standardise clinical practice by using the same terms, defined by the ICS [15]. It could be argued that this method is not ideal, as it does not reflect daily life, is invasive and is performed in an artificial environment. Results can also depend on how patients understand instructions from the investigator and how co-operative they are [10].

On the other hand, the diary is a good method for studying bladder behaviour in daily life. The SR-BD is cost-effective, non-invasive and could identify patients with a small bladder capacity, those with increased water or caffeine intake, or those who prefer to void frequently to avoid leakage. It can also be used to assess treatment, for example, if a beneficial effect of the use of anti-muscarinics needs to be assessed by looking at a reduction in the episodes of incontinence or urgency [30]. A diary without a bladder-sensation assessment included has more limited diagnostic power [6]. Other investigators think that the SR-BD only provides information about the sensation before the void and does not show how the bladder sensation develops [31], which means that the utility of this tool is still debatable. Equally, the duration of the bladder diary may affect its reliability.

Bladder diaries and cystometry measure bladder sensation as episodic events, but it is now known that the perception of the bladder is more of a continuous process. Using other methods had corroborated this: VAS [12, 29], and focus groups [28, 29]. The VAS evaluates bladder sensation continuously, from an empty to a full bladder. It helps to know the degree of sensation before each desire, but also gives information about how the sensation develops. The VAS gives a more precise assessment of the desire to void during bladder filling than any other method. Some could argue that as the patient needs to focus on sensation during filling, the results may not correspond to daily life symptoms.

Combined with a forced diuresis protocol, the VAS seems to be an excellent tool for assessing bladder sensation. It is non-invasive and measures its perception in a continuous manner. Although forced diuresis is likely to be more physiological than cystometry, this statement could be debatable, as this protocol induces diuresis that is different than in daily life [26, 29].

## Conclusions

Over the last few years, there has been much interest in finding the most valid and reliable method for measuring bladder perception. Unfortunately, despite all efforts, there is no conclusion about which tool to use. Given that bladder filling is a continuous and increasing perception, we suggest that a continuous method for recording it should be used. Although a forced diuresis protocol is both physiological and records sensation continuously, it is unlikely to be useful in clinical diagnosis. At present, there is limited research using the tool to study bladder sensation in different disease states.

## Abbreviations

FDV: First desire to void
FSF: First sensation of filling
ICC: Intraclass correlation
ICS: International Continence Society
LUTS: Lower urinary tract symptoms
MCC: Maximum cystometric capacity
OAB: Overactive bladder
SDV: Strong desire to void
SR-BD: Sensation-related bladder diary
UDV: Urgent desire to void
VAS: Visual analogue scale
